# 

**Published:** 1864-10

**Authors:** 


### DENTAL REGISTER

### OF THE WEST.

EDITED BY

J. TAFT AND GEO. WATT.

OCTOBER,–1864.

MONTHLY:

Three Dollars per Annum, in advance.

CINCINNATI:

J. TAFT, PUBLISHER.

J. Taft, Dentist, 172 Race Street.

